# Headache characteristics in COVID-19 pandemic-a survey study

**DOI:** 10.1186/s10194-020-01188-1

**Published:** 2020-10-13

**Authors:** Özge Uygun, Mustafa Ertaş, Esme Ekizoğlu, Hayrunnisa Bolay, Aynur Özge, Elif Kocasoy Orhan, Arif Atahan Çağatay, Betül Baykan

**Affiliations:** 1 Department of Neurology, Istanbul Faculty of Medicine, Istanbul University, Çapa, Istanbul, Turkey; 2 Department of Neurology and Algology, Gazi University Faculty of Medicine, Besevler, 06510 Ankara, Turkey; 3 Department of Neurology, Mersin University School of Medicine, Mersin, Turkey; 4 Department of Infectious Diseases and Clinical Microbiology, Istanbul Faculty of Medicine, Istanbul University, Çapa, Istanbul, Turkey

**Keywords:** COVID-19 infection, Headache, Migraine

## Abstract

**Background:**

Headache is the most common COVID-19-related neurological symptom. We aimed to reveal diagnostic clues of headache for COVID-19 infection and to investigate the course of primary headaches during the pandemic.

**Methods:**

We developed a detailed web-based questionnaire screening the characteristics and course of headaches besides clinical COVID-19 features. The participants were grouped according to being diagnosed with COVID-19 infection or not, and having previous or new-onset headaches. The COVID-19 related headache features and their associations with other clinical features were investigated. A binary logistic regression model was performed to differentiate the characteristics of headache related to COVID-19.

**Findings:**

A total of 3458 participants (2341 females;67.7%, 1495 healthcare workers;43.2%) with a mean age of 43.21 ± 11.2 years contributed to the survey. Among them, 262 participants had COVID-19 diagnosis and 126 (48.1%) were male. The rate of males in the group without COVID-19 was 31% (991 out of 3196 participants) showing significant gender difference between groups (*p* < 0.000). COVID-19 related headaches were more closely associated with anosmia/ageusia and gastrointestinal complaints (*p* < 0.000 and *p* < 0.000), and showed different characteristics like pulsating, pressing, and even stabbing quality. Logistic regression analyses showed that bilateral headache, duration over 72 h, analgesic resistance and having male gender were significant variables to differentiate COVID-19 positive patients from those without COVID-19 (*p* = 0.04 for long duration and *p* < 0.000 for others). A worsening of previous primary headaches due to the pandemic-related problems was not reported in the majority of patients.

**Interpretation:**

Bilateral, long-lasting headaches, resistance to analgesics and having male gender were more frequent in people with COVID-19 in conjunction with anosmia/ageusia and gastrointestinal complaints. These features may be helpful for diagnosing the headache related to COVID-19 during the pandemic.

## Introduction

Coronavirus disease-19 (COVID-19) caused by the severe acute respiratory syndrome coronavirus 2 (SARS-COV-2) first emerged in Wuhan towards the end of 2019 [1]. The pandemic is now influencing the whole world with a large number of deaths, besides many other medical and social consequences. The most common clinical picture of COVID-19 is characterized by manifestations of the respiratory system, as the name of the virus implies. However, many other complaints such as anosmia, ageusia, diarrhea and also headache have been noted in the clinical spectrum, with an increasing number of patients [2]. A handful of reports disclosed that headache is among the COVID-19-related symptoms, showing highly variable rates across the studies [3–6].

There are some studies and reviews highlighting that the most common neurological symptom is headache, often accompanied by high fever, moreover headache can occasionally be seen alone as the first sign of the disease [2, 7]. A recent meta-analysis reported that the prevalence of headache was 10.9% with a high level of heterogeneity [8]. Therefore, detailed questioning of the presence of headaches in patients who are admitted to the emergency and outpatient departments is still an important step in the prompt recognition of the infection in some patients.

In clinical observations and small case series, COVID-19 related headache was described as acute at onset, usually occurring in a different character unlike previous headaches [9, 10]. However, there are no systematic data on the headache characteristics.

Another important problem is the situation of patients with previous severe headaches like migraine in the COVID-19 pandemic era. COVID-19 itself or its psycho-sociological effects may cause more headache burden, along with the problems of quarantine and these points are not yet investigated thoroughly.

In this study, we aimed to reveal COVID-19 related headache characteristics and its associations. Our secondary aims were to investigate the features and course of pre-existing headaches as well as new-onset ones in participants without COVID-19 but having headaches during the pandemic.

## Methods

### Design of a web-based questionnaire

After the emergence of COVID-19 for the first time in March 2020 in our country, the complaints of peculiar headaches in consulted cases and some distinctive headache features in admitted patients attracted our attention. For this reason, a detailed questionnaire was developed focusing on headache characteristics, besides some clinical COVID-19 features by a panel of headache experts, after rounds of online discussions.

The number of the questions was tried to be kept in an acceptable limit to optimize the attention during answering the survey and the survey was tested before its submission by five patients for clarity of the questions. The web-based, user-friendly technical design was planned by the experts of the Istanbul University Department of Informatics.

The features of previous headaches were screened and headache diagnosis was made according to International Classification of Headache Disorders, 3rd version (ICHD-3 criteria) [11]. Possible changes in the headache course during the pandemic along with perceived precipitating factors and related characteristics were queried with scrutiny. Important characteristics such as the duration, frequency, course, localization, the severity of headaches, and treatment response were asked separately before and during the COVID-19 pandemic. The questionnaire has also asked if the participant had been diagnosed with COVID-19 as well as the symptoms of COVID-19 infection in detail for relevant patients. Even if the patient was not diagnosed with COVID-19, factors secondary to the pandemic lifestyle as possible triggers of headache, such as wearing a mask, having the fear of infection, etc. were evaluated. The questionnaire has included a total of 39 questions, and based on four different parts, in terms of demographics, previous headache features, reported COVID-19 infection features, and details of the headache course after the pandemic.

The participants were invited by means of social media (using Twitter, Instagram or Whatsapp) using a web-based link, suitable also for smart phones, after the Ethics Committee approval (17.04.2020/520). Discharged COVID-19 patients of the Istanbul University Faculty of Medicine Hospital were invited by text messages to volunteer the survey, in order to increase the number of patients with COVID-19 infection-related headache.

### Inclusion and exclusion criteria

**Fig. 1:**
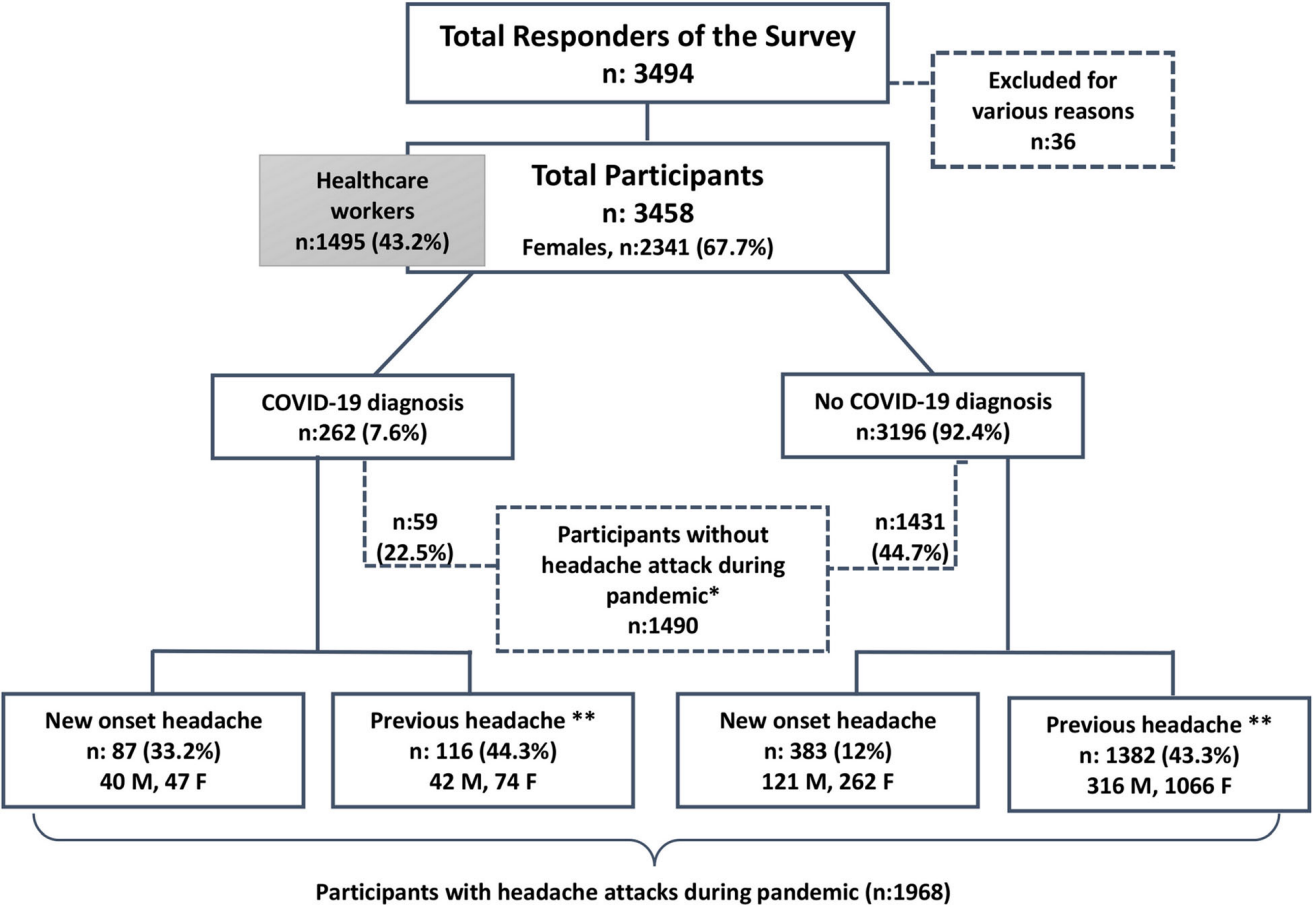
Flowchart of the study. *Please note that some participants with previous headaches did not experience headache attacks during the pandemic (March–April 2020). **Only those participants with headache attacks during the pandemic could be included in the analyses, done in terms of before-after pandemic comparisons. M; male, F; female

Eligible participants older than 18-years who had a COVID-19 infection experiencing headache among their symptoms or who had any type of headache before the pandemic, or developed new-onset headaches were included. The volunteers who participated in the survey were grouped according to being diagnosed with COVID-19 infection or not. In our country’s protocol, only those patients who have polymerase chain reaction (PCR) positivity, are diagnosed with COVID-19. Especially, healthcare workers were encouraged to attend the study. Figure 1 shows the flowchart of our study. Surveys completed by participants under age of 18 or those reporting inconsistent responses were excluded. Participants without headache during the pandemic were not included in the statistical analysis.

### Study period

The duration of survey implementation was planned as 15 days, starting on 1 May 2020. Although reaching a high number in a few hours, the survey was continued to the end of this period to include more patients with COVID-19 related headaches.

### Statistical analysis

Descriptive analyses were applied for four main groups of participants (Fig. 1, Table 1); the participants with or without COVID-19 were compared in relation to the presence or absence of previous headaches before the pandemic of COVID-19 in regard to headache characteristics by the chi-square test and t-test where appropriate. A binary logistic regression model was performed to explore the differentiating headache variables between COVID-19 positive and negative cases. The odds ratios were calculated for significant infection-related features, such as anosmia/ageusia and gastrointestinal complaints like diarrhea. IBM SPSS Statistics Version 22 was used and *p* < 0.05 was considered as statistically significant.

## Results

A total of 3458 participants (2341 females) contributed to our survey. Of this main group with and without headache during the pandemic, 262 patients were diagnosed with COVID-19 by PCR (Fig. 1). These 262 patients were grouped as COVID-19 positive including 136 females (51.9%) and 89 healthcare workers (9.8%).

### The characteristics of patients with COVID-19 infection

In the COVID-19 positive group, the rate of males was 48.1% (126 out of 262 patients), whereas in the COVID-19 negative group this rate was 31% (991 out of 3196 participants), showing a significant gender difference (*p* < 0.000). Headaches lasting over 72 h were reported by 10.3% (27 out of 262 participants) of COVID-19 infected patients versus 4.1% (130 out of 3196 participants) of the COVID-19 negative group (*p* < 0.000).

In this study, 1968 participants with or without COVID-19 infection reported headache attacks during the pandemic. Among them 714 (36.3%) had migraine and 1077 (54.7%) participants were diagnosed with tension type headache (TTH) according to ICHD-3 criteria. The headache characteristics, accompanying features, treatment responses of these patients experiencing headache during the pandemic, according to having or not having prior headaches were shown in Table 1, comparatively. Osmophobia was more frequently reported in the group with COVID-19. Most of the COVID-19 cases (71%) experienced nausea and gastrointestinal symptoms, for example, diarrhea/stomachache were present in more than half of them. Interestingly, pulsating quality was more pronounced in patients with previous headaches in the group with COVID-19 in comparison to the group with history of new onset headache.

**Table 1:** Demographic features of participants and characteristics of headache experienced during the pandemic period

(*n* = 1968)	COVID-19 positive^a^			No COVID-19^b^				
	Prior headache (*n* = 116)	No prior headache (*n* = 87)	p	Prior headache (*n* = 138)	No prior headache (*n* = 383)		p (1–3)†	p (2–4)‡
Age year,mean (SD)	39.7 (11)	38.2 (11)	.370	41.0 (10)	42.2 (11)	.061	.175	.004
Sex (male %)	36.2	46.0	.104	22.9	31.6	.000	.001	.008
Healthcare worker (%)	44.0	43.7	.541	46.1	48.3	.239	.366	.255
Headache duration hours mean (SD)	52.1 (57)	45.3 (53)	.430	38.4 (53)	45.9 (63)	.039	.014	.949
Headache characteristics								
Pulsating %	50.9	32.5	.008	55.5	42.5	.000	.202	.064
Pressing %	31.9	43.7	.058	29.0	38.6	.000	.289	.227
Fiery %	2.6	2.3	.633	2.7	5.0	.022	.625	.219
Stabbing %	12.9	16.1	.330	11.1	11.2	.497	.315	.142
Accompanying symptoms								
Nausea %	70.8	71.3	.538	53.5	52.9	.450	.000	.002
Phonophobia %	67.0	67.9	.509	71.2	66.6	.056	.198	.465
Photophobia %	63.9	63.0	.508	60.5	51.2	.002	.276	.038
Osmophobia %	39.0	50.0	.090	30.4	28.5	.292	.044	.000
Allodynia %	31.9	–	–	41.8	–	–	.024	–
Anosmia/ageusia %	74.5	73.2	.479	19.1	23.1	.073	.000	.000
Sore throat, rhinorrhea %	62.2	70.4	.152	41.8	54.4	.000	.000	.006
Stinging, burning, tearing in the eyes	63.2	64.6	.481	44.8	49.8	.063	.000	.011
Use of analgesics %	84.3	79.8	.257	82.8	69.8	.000	.389	.042
No response to analgesics %	16.5	22.4	.227	7.1	8.0	.353	.002	.002
Partial response to analgesics < 50%) %	38.1	38.8	.530	25.5	37.6	.000	.006	.484
Improved with analgesics > 50%	32.0	25.4	.231	26.1	27.4	.362	.129	.436
Completely recovered with analgesics %	12.4	11.9	.568	39.2	26.2	.000	.000	.008

^a^ Diagnosed by positive PCR test; ^b^ No COVID-19 symptoms or diagnosis; †**p (1–3)** *p* value between the patients with prior headache with or without positive COVID-19 test; ‡**p (2–4)** *p* value between the patients with no prior headache with or without positive COVID-19 test (significant test values are marked as bold)

The great majority of COVID-19 patients with previous headaches (79.5%, 89 out of 112 responders) reported that their new emerging headaches during the infection period were different from their usual headaches; among them, 50% (56 participants) disclosed that this new headache was totally different, whereas 29.5% (33 participants) reported some differences despite some similar properties resembling previous headache features. On the other hand, among the participants without COVID-19 diagnosis but with previous headaches, 62.7% (785 out of 1252 responders) disclosed that their headaches were identical to the pre-existing episodes. Among them, only 13.9% (174 participants) reported entirely different attacks, whereas 23.4% (293 participants) reported partly different headaches.

Severity of the previous headaches did not relate to receiving COVID-19 diagnosis; COVID-19 positive patients had reported headaches of mild intensity in 26.6% (34 out of 128 responders), moderate-intensity in 47.7% (61 participants), severe in 23.4% and very severe (dependent to bed/hospital) in 2.3% (3 participants). The corresponding rates of headache intensity were 27.7% (484 out of 1747 responders), 46.7% (816 participants), 21% (367 participants), and 4.6% (80 participants) in COVID-19 negative participants.

Among all 262 patients diagnosed with COVID-19, 40% (104 participants) reported high fever (over 38 °C), 49.2% (128 participants) prominent cough, 48.3% (125 participants) sore throat and 33.5% (87 participants) shortness of breath. On the other hand, diarrhea/stomachache was present in 57.7% (150 participants) and lastly anosmia/ageusia was present in 60.4% (157 participants) of this group. There were 59 (22.5%) COVID-19 positive patients who had previous headaches, who did not experience headaches during the pandemic period of 2 months at the time of their participation in the survey. At least one close contact with the disease was present in 65.4% (170 participants) of the COVID-19 positive patients and 90.1% of the patients lived under quarantine.

### Triggers of headache in the study population

**Fig. 2:**
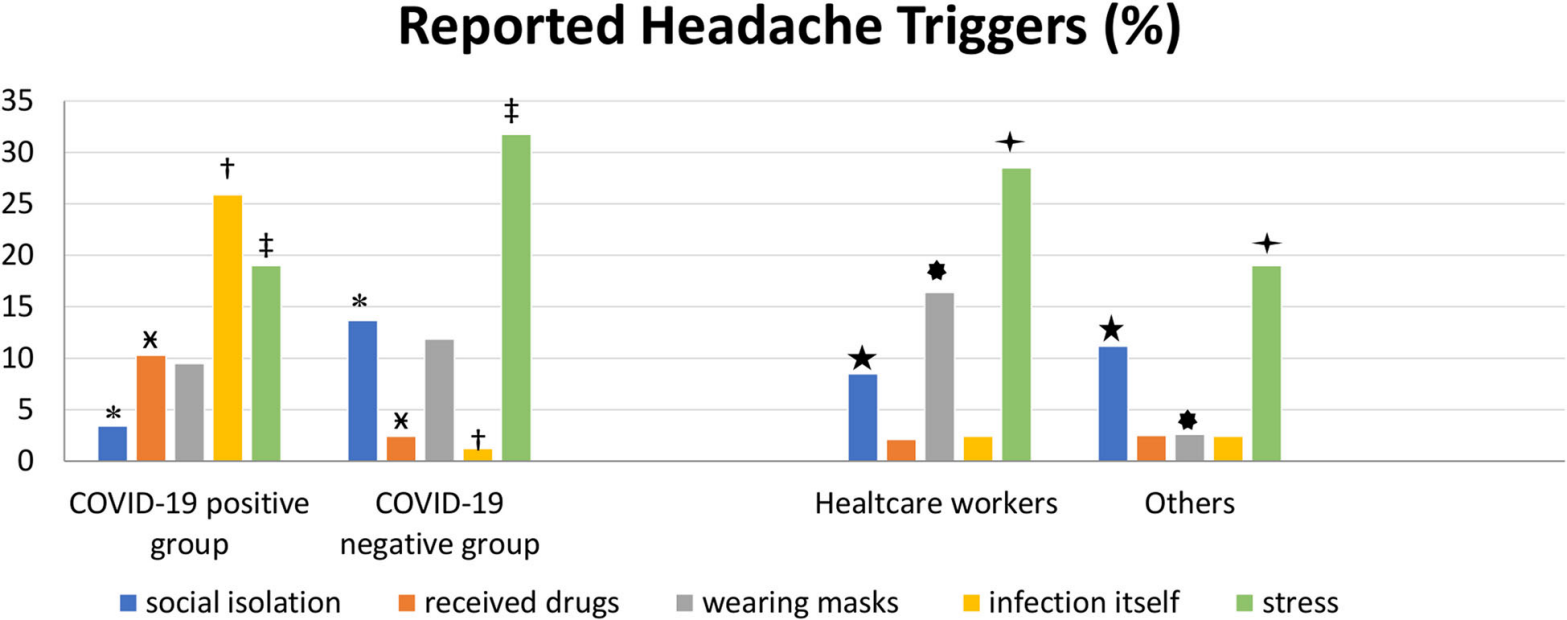
Reported triggers for headache were shown comparatively in COVID-19 positive and negative cases. The second part of the figure shows the triggers reported by healthcare workers compared to the others. Legend. *p:0.0006, ^ӿ^p:0.0002, ^†^p:0.0001, ^‡^p:0.0015 showing statistically significant differences between each reported trigger in COVID-19 positive and negative patients; and ★p:0.049, ✸p:0.0001, ✦p:0.0001showing statistically significant difference between healthcare workers and others, with Pearson Chi Square Test

The triggers of headache reported by the participants are shown comparatively in COVID-19 positive and negative cases in the Fig. 2; the second part of this figure shows the triggers reported by healthcare workers compared to the others. We noticed that stress was the most common trigger up to 30% of the participants as seen as in the Fig. 2 and social isolation triggered headache noteworthy in patients without COVID-19. On the other hand, the patients diagnosed with COVID-19 reported also infection itself and the drugs as triggers of headache.

### The course of headache in participants during the pandemic

**Fig. 3:**
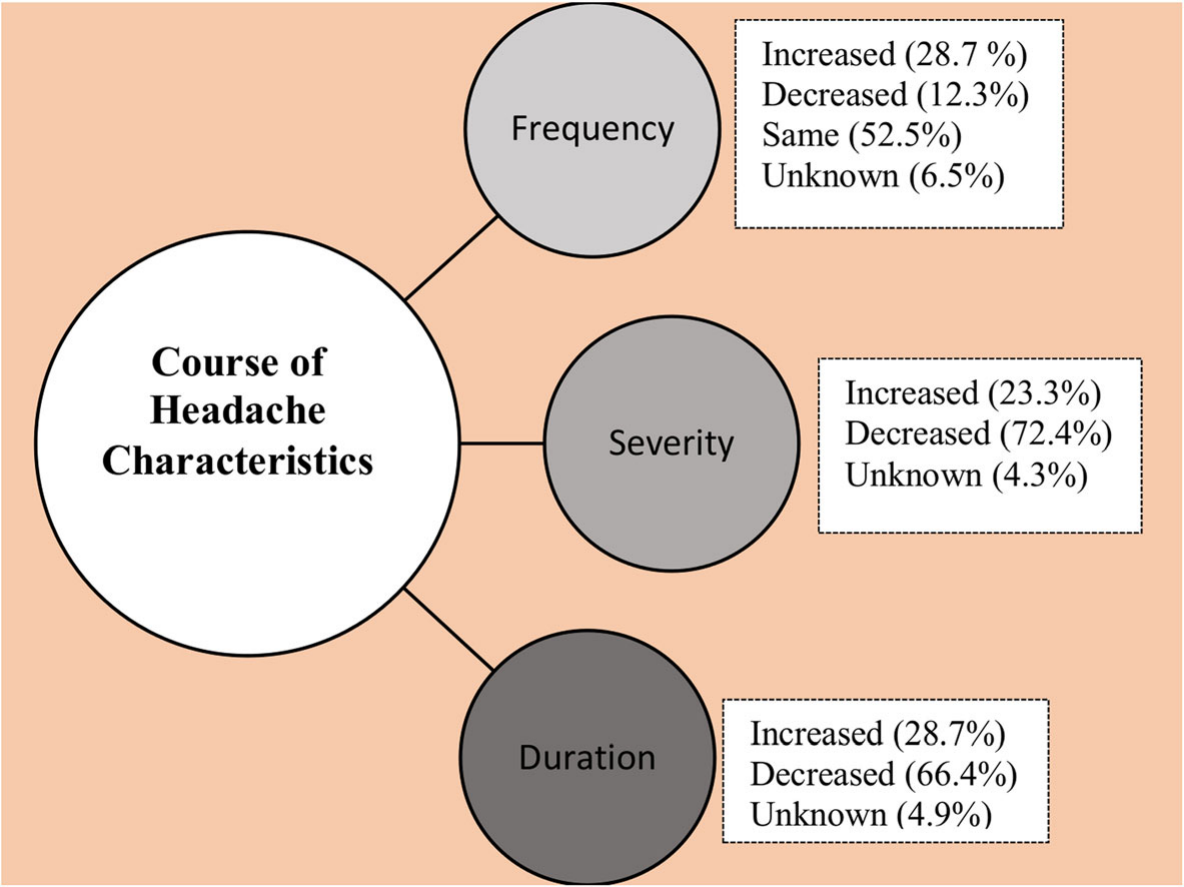
The course of headache characteristics

The reported changes in the headache course after the pandemic were shown in Fig. 3. Among the responding 1886 patients, 23.3% reported an increase in severity of headache, 28.7% reported an increase of the headache duration, and lastly 14% reported deterioration in the accompanying symptoms. The decreased headache frequency in 12.3% of the group was remarkable.

Our analysis showed that among the patients with pre-existing migraine diagnosis, the frequency of pulsating headache character decreased from 60.5% (328 out of 542 pts) to 55% (22 out of 40 pts) in those receiving COVID-19 diagnosis. However, this latter rate was still significantly higher in comparison to 34.1% (31 out of 91 pts) compared to COVID-19 positive patients without previous headache (*p* = 0.033).

The worsening of previous headache and analgesic unresponsiveness did not show any difference in relation to ages of the participants with COVID-19.

### Differentiating variables of COVID-19 related headache

**Fig. 4:**
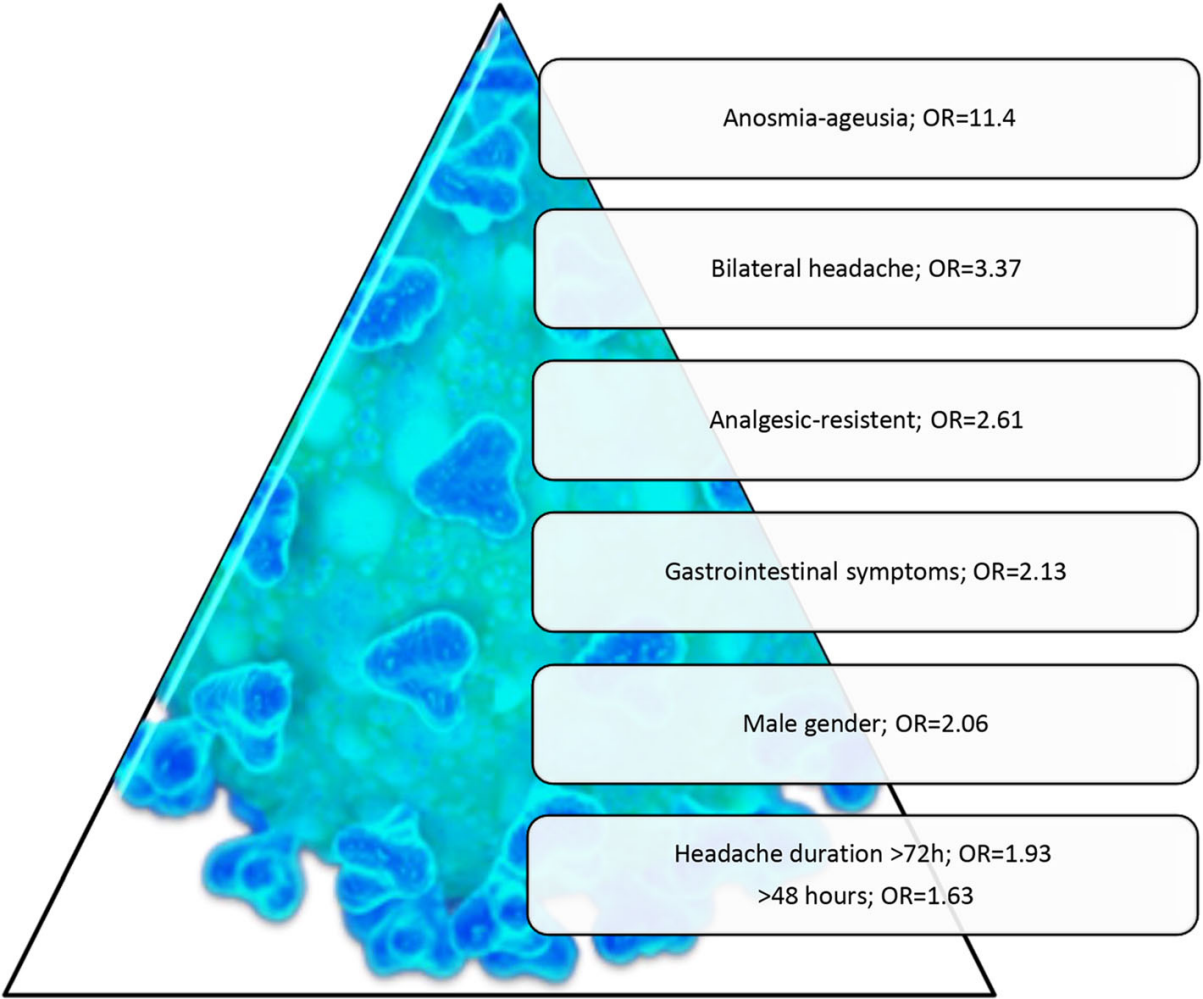
Odds ratios of differentiating variables related to the presence of headache in patients with COVID-19 diagnosis reporting headache during the pandemic

The important differentiating variables for COVID-19 infection patients suffering from headaches were summarized in Table 2 and graphed as Fig. 4, with calculated Odds ratios (OR). According to our findings, the presence of bilateral headache, duration over 72 h, male gender, analgesic resistance, gastrointestinal symptoms and anosmia/ageusia showed increased risk of having headache related to COVID-19 infection.

**Table 2:** Odds ratios of differentiating variables of the headache in patients with COVID-19 diagnosis

Headache characteristics	COVID-19 positive n (%)	No COVID-19 n (%)	OR
Presence of anosmia/ageusia	142(74%)	282(%20)	11.4
Bilateral headache	166 (85%)	994 (64%)	3.37
Analgesic-resistant	32(%18)	116(8%)	2.61
Gastrointestinal symptoms	137(70%)	808(53%)	2.13
Male gender	82(%40)	437(25%)	2.06
Headache duration > 72 h	27(13.3%)	130(7%)	1.93
Headache duration > 48 h	55(27%)	328(18.5%)	1.63

Additionally, binary logistic regression was computed with significant headache variables which could be observed in everyday practice to distinguish COVID-19 related headaches. The model summary showed a − 2 Log likelihood ratio of 1003.6 (Nagelkerke R Square: 0.105) and using this model 88.6% of the COVID-19 patients could be classified correctly with a cut off value of 0.5. We did not enter infection-related features in this analysis, because they are clear clues for COVID-19 and do not appear in control cases. Our findings disclosed that bilateral headache, duration over 72 h, male gender, and analgesic resistance were important variables to differentiate between COVID-19 positive patients from negative ones, showing statistical levels as *p* = 0.04 for long duration of headaches and *p* < 0.000 for the remaining variables (Supplementary Table 1).

## Discussion

This first careful analysis of emerging headache characteristics in the pandemic showed that COVID-19 related headaches are more closely associated with anosmia/ageusia and gastrointestinal complaints, in comparison to other usual infection findings. Moreover, bilateral headache, duration over 72 h, male gender, and analgesic resistance are highly important variables to differentiate between COVID-19 positive patients from negative ones. Although we expected prominent worsening of primary headaches due to the pandemic-related problems, this happened only for less than 1/3 of participants, mostly related to stress.

### Gender difference of COVID-19 related headaches

Despite the well-known predominance of headaches in females and the fact that more than 2/3 of participants answering our survey consisted of females, COVID-19 related headaches were reported by male patients at a high rate. This interesting finding is somewhat in line with the predominance of COVID-19 in male gender, with changing reported rates around 56–73% [12–14]. It could be hypothesized that this reversed gender dominance may relate to comorbidities like atherosclerosis and hypertension which are more frequent in males. However, females with nearly three times higher rates of migraine could still have outnumbered in COVID-19 related headaches [12, 15]. Therefore, this finding is a striking point that needs further careful elaboration. Given the higher risk of male gender in COVID-19 cases, a protective role of female hormones or of the location of Angiotensin-converting enzyme 2 (ACE-2) on the X chromosome could be speculated [9, 12]. Moreover, ACE-2 expression level which is critical for the SARS- CoV-2 entry to the cells was found different between genders [14]. There is some evidence that immune activity is more efficient in females in other viral infections [16]. It is tempting to speculate that SARS-CoV-2 may trigger some silenced genes related to innate immunity in the X chromosome, so two X chromosomes may serve for a more effective and balanced war against COVID-19 related hyperactivation of immune pathways. Moreover, estrogens and progesterone have anti-inflammatory actions partially through inflammasome activation in some models [17]. ACE also affects the display of major histocompatibility complex (MHC) class I and MHC class II peptides [18]. Thus, further work with these gender-related differences may give us some clues to find out novel protective ways against COVID-19.

### Possible mechanisms underlying COVID-19 related headaches in the light of our findings

The underlying mechanisms of headache related to COVID-19 are not clear at this early moment [9]. A direct invasion of trigeminal nerve endings in the nasal or oral cavity by the virus seems one of the most reasonable mechanisms underlying headache according to our results showing the close relation between headache and anosmia/ageusia. Some coronaviruses were shown to be neurotropic and former SARS-CoV has been observed in the human brain [19, 20]. Therefore, it is highly likely that SARS-CoV-2 may also enter the nervous system via the cranial nerves. Since the first observations in China, there are many worldwide reports showing heterogeneous prevalence figures around 5–85% of loss of smell [21, 22]. These profound differences may relate to the viral load differences and a different individual immune response between younger milder symptomatic outpatients, who are eager to report their symptoms, and contrarily severe COVID-19 inpatients with prominent respiratory problems who probably under-report this relatively milder problem. It is well-known that methodological differences exist between questionnaire studies and objective measurements, the former being mostly with lower prevalence. Despite this fact, the rate of anosmia/ageusia was high in our study. These two distinct problems caused by different nerves were not easy to differentiate from each other in daily life, therefore anosmia and ageusia were evaluated together in our study. It was remarkable that nasal obstruction and rhinorrhea were frequently reported but not strictly correlated with anosmia and ageusia according to our results. Although the trans-synaptic transfer of SARS-COV-2 is not proven yet, this possibility of the trans-synaptic route was documented for other coronaviruses [23]. Entrance from the nasal cavity to the olfactory bulb, then spreading to the brainstem via the piriform cortex with both passive diffusion and axonal transport has been demonstrated [24].

Besides the described headache characteristics and COVID-19 related respiratory tract symptoms, abdominal pain and diarrhea should be taken into account to evaluate these patients [3–5, 25]. Our study found a high rate of gastrointestinal symptoms like diarrhea/stomachache in more than half of the COVID-19 cases and also high rates of nausea (71%) as an accompanying symptom of headache. A previous interesting report from China on non-classical symptoms indicated that 21.6% of patients had gastrointestinal symptoms, associated with headache, which is a higher number in comparison to patients without gastrointestinal symptoms [26]. The intriguing relationship of headache with gastrointestinal symptoms also brings to mind several interesting mechanisms including increased circulating Calcium gene related peptide (CGRP) levels and “gut-brain axis” concept where several inflammatory mediators like Interleukin-1β (IL-1 β), Interleukin-6 (IL-6), Interleukin-8 (IL-8), and Tumor necrosis factor-α (TNF-α), besides gut microbiota, and neuropeptides including CGRP are thought to play a role in this interaction. Another relevant consideration is systemic CGRP increase, possibly induced by both angiotensin II and IL-6 levels, as CGRP is clearly associated with trigemino vascular activation resulting in headache, increased gastrointestinal (GI) motility leading to diarrhea, further triggering inflammation and vascular edema [9, 27]. Taken together with the unusually high rate of ageusia-anosmia (60.4%), this data may lead us to the footsteps of the viral pathway in the brain. Thus, we may also suggest that neuronal invasion of this new coronavirus may cause the dysfunction of the network at brainstem sites, in addition to headache. Nausea and vomiting are associated GI symptoms of migraine headache, yet the diarrhea is a distinct GI feature associated with headache in COVID-19, which clearly shows that opposite influencers play role in SARS-CoV-2 infected gut and trigeminal nerve.

Headache in relation with a systemic viral infection, (without signs of meningo-encephalitis) is described in the International Classification of Headache Disorders-3. The underlying mechanisms of this entity are not illuminated so far. Regarding its characteristics, diffuse pain of moderate/severe intensity, commonly with fever was noted [11]. However, in our analyses, the association of headache with fever seems not to be decisive (in all COVID-19 patients with headache, only 40% reported high fever). Also, rhinosinusitis and other respiratory tract symptoms did not seem to explain the headaches in many of these cases, as seen in Table 1. Therefore, for this emerging COVID-19 related headache, the simplistic view of a “causal” relationship with fever or upper respiratory symptoms is not explanatory.

### The course of headaches during the pandemic and reported triggers for headache

Most of the patients with pre-existing headaches easily noticed that this was a different problem if they had COVID-19 related headache according to our survey. In a recent case report, the authors highlighted the need to consider secondary headaches, related to central nervous system infections in the setting of COVID-19 in patients experiencing refractory headache, even if the patient had chronic migraine [28]. On the other hand, it was also intriguing that 22.5% of the COVID-19 positive cases with previous headaches did not suffer from headache during the pandemic and during the infection. There is no clear explanation for the lack of headache in these cases; causes related to viral load, transmission route, or individual differences may play a role. Other interesting data were the stabile course (53%) or even decrease of the attack frequencies (12%) and reduced severity of the pre-existing headaches in the pandemic period despite the apparent stressful conditions. Social isolation may have helped to avoid stressful social interactions; a healthy diet, and mild sports activities are possible with spending more time at home, also reducing the stress of daily-work life during the pandemic; all these points were possible reasons for the better headache outcome than expected.

The triggers for headaches showed significant differences between participants with and without COVID-19 infection. Headache was triggered more frequently by stress and social isolation in patients without COVID-19 whereas patients diagnosed with COVID-19 reported also infection itself and the drugs as triggers of headache. Furthermore, wearing masks as well as stress both were the main triggers of headache and were more frequently reported by healthcare workers than by the others. Standard hygiene precautions seemed to reduce the risk for the healthcare workers who did not show an increased risk for COVID-19 compared to other participants in our study. It was reported from Germany that an overall seroprevalence of SARS-CoV-2 in a tertiary hospital was low, given the standard hygiene measures taken [29].

### Other differentiating features and associations of COVID-19 related headache

The clinical features of COVID-19 infection in non-hospitalized adults were reported to be different from hospitalized patients warranting greater awareness of this wider spectrum of clinical symptoms [30]. Headache as the leader of the COVID-19 related neurological symptoms is the most frequent complaint in outpatient clinics [31]. Thus, it is essential to recognize those patients with COVID-19 at the beginning of the visit or even in telemedicine visits. In this study, we provided evidence that long-lasting bilateral headaches over 48–72 h and headaches resistant to analgesics suggested the likelihood of being infected by COVID-19 similar to other secondary headaches. The interesting distributions of pulsating and pressing characters in COVID-19 patients, as seen in Table 1, showed that pulsating type was more pronounced in patients with previous headaches; this may indicate that individual backgrounds are important in the final phenotypic presentation of COVID-related headache. COVID-19 infection may play a synergistic role in nociception using similar pathways of the trigeminovascular complex as the underlying primary headache such as migraine. However, different characteristics like pulsating, pressing, and even stabbing quality may indicate that more than one mechanism is involved in COVID-19 related headache emergence. A case report by a headache expert diagnosed with COVID-19 also indicated that several types of headaches can be seen during COVID-19 infection based on a single case (himself) [32]. Our data had a cross-sectional design; and many participants have chosen only one type despite the availability of multiple choices. Another intriguing finding was that photophobia was more frequently experienced by infected participants than those without COVID-19, reaching statistical significance between the subgroups without prior headache. Moreover, osmophobia was more frequently seen in the group with COVID-19, which may also be related to the olfactory dysfunction. Further basic studies are needed to clarify the mechanism of COVID-19-related headache and to unravel the mysteries of the environmental factors including viruses for the headache mechanisms, to make the pandemic disaster an opportunity.

### Limitations and strengths of the study

There are some limitations to this study. First of all, we investigated headache characteristics via questionnaire; the results were based on the answers of the patients, with the potential of a reporting bias well known in all survey studies. Secondly, the patients with COVID-19 were not examined by a physician or headache specialist. Furthermore, our questionnaire was a web-based survey, therefore, only individuals who were able to use new technological devices, thus probably younger and educated people could participate in the study. Among the participants, there may also be some patients who were not tested for COVID-19 due to the lack of other accompanying symptoms. Moreover, patients with severe COVID-19, at the time of the survey, could not be included.

The main strength of our study was the participation of a large number of people in a very short time in the increasing phase of the pandemic. We used a detailed dedicated questionnaire investigating various characteristics of previous and current headaches, including also cross-questions to avoid misunderstandings. The answers given by participants were also examined meticulously to minimize discrepancies. Furthermore, the presence of healthcare workers, reaching nearly half of the participants has increased the reliability of the study.

## Conclusion

The COVID-19 pandemic seems to have a particular effect on the characteristics and the course of headaches in individuals with and without COVID-19 diagnosis, according to our findings. We disclosed that having male gender, bilateral, long-lasting headaches, and resistance to analgesics were more frequently seen in people with COVID-19 infection in conjunction with anosmia/ageusia and gastrointestinal complaints, besides other infection findings. We propose that these features may be diagnostic for COVID-19 infection in the clinical evaluation of headache patients during the pandemic. We think that COVID-19 related headache should be considered as a separate entity, among the infection-related secondary headaches, with this different profile.

## Supplementary information

**Additional file 1: Supplementary Table.** Logistic regression analysis model to differentiate patients with COVID-19 from those without COVID-19 based on headache characteristics.

## Data Availability

This study is a web-based questionnaire, the data is not applicable.

## Abbreviations

COVID-19: Coronavirus disease 2019
SARS-CoV 2: Severe acute respiratory syndrome coronavirus 2
ACE-2: Angiotensin converting enzyme
TNF- α: Tumor necrosis factor-α
IL-1β: Interleukin-1β
IL-6: Interleukin-6
IL-8: Interleukin-8
CGRP: Calcium gene related peptide
TTH: Tension type headache
ICD-3: International Classification of Headache Disorders, 3rd Edition
PCR: Polymerase chain reaction
MHC: Major histocompatibility complex
RR: Relative risk ratio
OR: Odds ratio
GI: Gastrointestinal
