# Supplementary material for: Headache characteristics in COVID-19 pandemic-a survey study
Source: J Headache Pain. 2020 Oct 13;21(1):121. doi: 10.1186/s10194-020-01188-1 (PMC7552597; doi:10.1186/s10194-020-01188-1)
Supplement: Supplementary file 1 — Additional file 1: Supplementary Table. Logistic regression analysis model to differentiate patients with COVID-19 from those without COVID-19 based on headache characteristics. [file 10194_2020_1188_MOESM1_ESM.docx]

**Supplementary Table: Logistic regression analysis model to differentiate patients with COVID-19 from those without COVID-19 based on headache characteristics**

| Variables |  |  |  |  |  | Exp(B) | 95% C.I. for EXP(B) | |
| --- | --- | --- | --- | --- | --- | --- | --- | --- |
|  | B | S.E | Wald | df | Sig. |  | Lower | Upper |
| Bilateral headache | -1.391 | 0.254 | 30.081 | 1 | 0.000 | 0.249 | 0.151 | 0.409 |
| Gender | -0.897 | 0.173 | 27.012 | 1 | 0.000 | 0.408 | 0.291 | 0.572 |
| Analgesic resistance | -1.005 | 0.236 | 18.153 | 1 | 0.000 | 0.366 | 0.231 | 0.581 |
| Duration>72 hours | -0.509 | 0.248 | 4.214 | 1 | 0.040 | 0.601 | 0.370 | 0.977 |
| Constant | 0.179 | 0.329 | 0.297 | 1 | 0.586 | 1.196 |  |  |

B: beta (regression coefficient); SE, standard error; d.f., degree for freedom; Sig., significance; Exp (B), OR: exponential B, odds ratio.
